# Two cases of multi-inflammatory syndrome in children (MIS-C) in adults in 2020

**DOI:** 10.1186/s12879-021-06911-0

**Published:** 2021-12-07

**Authors:** Zachary Ciochetto, Peter L. Havens, Sol Aldrete

**Affiliations:** 1 Division of Infectious Diseases, Medical College of Wisconsin Affiliated Hospitals, 8701 W Watertown Plank Rd, Milwaukee, WI 53226 USA; 2 Infectious Diseases, Children’s Hospital of Wisconsin, 999 North 92nd Street, Wauwatosa, WI 53226 USA

**Keywords:** SARS-CoV-2, COVID-19, Multi-inflammatory syndrome in children, Cardiac, Adults

## Abstract

**Background:**

Late complications and longer-lasting sequelae of COVID-19 infection in adults can occur. Cardiovascular involvement including reduced ejection fraction, coronary artery aneurysms, and pericardial involvement have been reported. Prompt recognition is the first step and secondly, these cardiovascular phenomena require an alternative set of therapeutics from the standard of care for acute COVID-19 infection.

**Case presentation:**

Here we describe two cases that fulfill the current case definition of the recently defined multisystem inflammatory syndrome in adults (MIS-A). One patient is a 27-year-old white female and the other a 21-year-old French creole male, both without any prior medical history. Both were hospitalized and found to have significant cardiac dysfunction and treated with IVIG, high dose aspirin, and corticosteroids with resolution of their acute illnesses and cardiac sequelae.

**Conclusion:**

Not only does the immediate impact of this viral infection need to be addressed, but also the long-term complications that could arise if not recognized and treated promptly as seen in our two cases. Patients can develop acute cardiovascular collapse and cardiogenic shock which requires high level of care and treatment within an intensive care unit. Depending on the complications, patients may require treatment for congestive heart failure, pericarditis, or even coronary artery disease acutely with close follow up to ensure improvement or resolution.

## Introduction

The clinical course of severe acute respiratory syndrome coronavirus 2 (SARS-CoV-2) infection in adults is characterized by both upper and lower respiratory tract symptoms and can include pneumonia and acute respiratory distress syndrome. In children, however, the primary respiratory illness may be absent or mild, with the most severe symptoms manifesting a few weeks after the primary infection as a post infectious inflammatory syndrome called multisystem inflammatory syndrome in children (MIS-C) [1]. MIS-C includes cardiac findings in up to 80% of affected children [2]. We report two cases of adults with no prior known history of cardiac disease hospitalized with COVID-19 infection manifesting clinically as MIS-C with significant cardiac involvement.

## Case presentation: patient 1

A healthy 27-year-old white female, without previous history of cardiovascular disease, presented to the emergency department (ED) with two days of fever, chills, chest pain, and shortness of breath. A month prior to her presentation she had been diagnosed with COVID-19 through PCR testing and had experienced mild upper respiratory symptoms which completely resolved within two weeks. Prior to returning to work she was retested for COVID-19 with two negative nasopharyngeal (NP) SARS-CoV-2 PCR tests 10 days prior to her current ED visit. In the ED, examination revealed blood pressure of 126/80 mm Hg, heart rate of 127 beats per minute, oxygen saturation of 97% while breathing room air and body temperature of 39.6 °C. A repeat NP swab was positive for SARS-CoV-2. Her chest radiograph showed a right basilar airspace opacity and she was admitted and started on empiric intravenous ceftriaxone and azithromycin.

Throughout her hospital stay, she had persistent tachycardia without tachypnea or gas exchange abnormalities, with oxygen saturation 95–96% on room air. Her physical examination revealed bilateral non-exudative conjunctivitis, mildly erythematous oropharynx without exudates, tachycardia, decreased breath sounds over the right lower lobe and a faint scattered macular rash on her inner thighs, abdomen, and upper back. Laboratory evaluation showed a total white blood cell count 9.4 × 10^9^/L (normal 4.5–12.0 × 10^9^/L), with absolute lymphopenia at 770 cells/L (normal 900–3200 cells/L) platelet count 137 × 10^9^/L (normal 150–450 × 10^9^/L), creatinine 0.73 mg/dL (normal 0.70–1.20 mg/dL), Ferritin 625.0 ng/ml (normal 10–120 ng/mL in females), CRP 25.10 mg/L (normal < 3.0 mg/L), and D-Dimer 1.63 mcg/mL (normal < 0.50 mcg/mL). Blood cultures, streptococcus pneumoniae urinary antigen, and legionella urine antigen-1 were negative. Computed tomography pulmonary angiography was negative for thrombi. Her fever resolved by hospital day 3 and antibiotics were stopped on day 5, but her tachycardia continued, and inflammatory markers continued to rise during that same time period (Ferritin to 1278 ng/mL; CRP to 30.2 mg/L; D-dimer to 2.45 mcg/mL). On her 4th hospital day, cardiac markers revealed an elevated troponin of 24 ng/L (normal < 10 ng/L) and markedly elevated pro-B natriuretic peptide of 5300 pg/mL (normal < 100 pg/mL). EKG showed sinus tachycardia with short PR and diffuse ST and T wave abnormalities. A transthoracic echocardiogram (TTE) revealed normal left ventricular (LV) dimensions, with LV systolic dysfunction with estimated LV ejection fraction (LVEF) of 47% with hypokinesis of mid distal anterior, anteroseptal, and apical inferoseptal walls.

Given her prior COVID-19 infection and current constellation of signs and symptoms (fever, persistent tachycardia, erythema multiforme-like rash, bilateral non exudative conjunctivitis), rising laboratory markers of inflammation and biochemical and TTE evidence of cardiac involvement, we were concerned for an MIS-C like condition. On her 5th hospital day, she received one dose of 650 mg of aspirin, and was started on intravenous immunoglobulin (IVIG 2 g/kg once) and tocilizumab (600 mg IV once) with significant improvement in clinical symptoms and resolution of tachycardia. Aspirin was not continued given the absence of coronary artery aneurysms. On hospital day 7 she was discharged to home on no medications, clinically well, with CRP down to 5.7 mg/dL. Six weeks after hospital admission, CRP was 0.4 mg/dL (normal) and echocardiogram demonstrated normal ejection fraction (LVEF 65%). Outside records of prior COVID antibody testing were not available. At the time of this hospitalization her SARS-CoV-2 PCR cycle threshold (CT) was 31.77 (cut off for detection CT < 40) suggesting low nasopharyngeal viral load. Given the history of initial pulmonary COVID-19 one month prior, the high CT (low nasopharyngeal viral load) during this hospitalization suggested the current illness represented late sequelae of the infection.

## Case presentation: patient 2

The second patient was a healthy 21-year-old French creole male with no significant past medical history who was transferred from an outside hospital for escalation of care due to COVID-19 infection and multi-organ failure. He had initially presented with eight days of subjective fever, headache, nausea, vomiting, abdominal pain, and shortness of breath. ED examination revealed blood pressure of 97/64 mm Hg, heart rate of 110 beats per minute, respiratory rate of 26 breaths per minute, oxygen saturation of 96% on room air and body temperature of 39.2 °C. A NP swab for SARS-CoV-2 was positive. Laboratory results showed a serum creatinine of 2.06 mg/dL, elevated liver enzymes (ALT 75 U/L, AST 47 U/L, direct bilirubin 3.9 mg/dL, total bilirubin 5.9 mg/dL, alkaline phosphatase 113 mg/dL), white blood cell count of 16.5 × 10^9^/L and platelet count of 173 × 10^9^/L. SARS-CoV-2 IgG antibodies were positive. His chest radiograph revealed patchy ground glass infiltrates in both lung fields. He was treated with IV ceftriaxone and azithromycin and switched to Vancomycin and Cefepime on hospital day 1.

His physical exam revealed bilateral conjunctival injection, clear lungs to auscultation bilaterally, diffusely tender abdomen without guarding or rash. Further evaluation showed elevated troponin of 782 ng/L, BNP 50,640 pg/mL, ferritin 671 ng/mL, D-Dimer 4.18, and CRP 29.20 mg/dL on initial presentation. EKG showed sinus tachycardia, with ST and T wave abnormalities in the anterolateral leads. His TTE revealed left ventricular systolic dysfunction with ejection fraction estimated at 50% and global hypokinesis. Given suspicion for MIS-C, he was given 650 mg of aspirin as well as 2 g/kg of IVIG over 12 h on day 2 of his hospital stay. The following day he was transferred to the intensive care unit for 24 h for vasopressor support with norepinephrine. It was around this time that his inflammatory markers peaked (ferritin 923 ng/mL, CRP 34.4 mg/dL). After receiving IVIG, aspirin, and resuscitation his symptoms improved, liver enzymes trended down, and his renal function came back down to his baseline after peaking at 4.13 mg/dL. His fever had initially resolved after day 2 but on day 5 he spiked another fever up to 38.9 °C and was started on steroids (prednisone 30 mg twice a day for five days followed by a 10-day taper) [3]. After initiation of steroids his fever did not recur. A repeat EKG prior to hospital discharge showed resolution of the ST changes seen on his initial EKG and repeat TTE is yet to be completed to check for normalization of his left ventricular systolic dysfunction and hypokinesis seen on his initial echocardiogram.

## Discussion and conclusions

MIS-C is a newly described rare but severe condition that has been reported approximately 2–4 weeks after the onset of COVID-19 in children and adolescents [4]. This disease process can have devastating consequences with high morbidity and mortality if not treated promptly. Originally thought only to affect children, this phenomenon is now being seen in adults. Patients must test positive for recurrent or recent SARS-CoV-2 infection by rapid PCR or antibody testing. Shock is a common feature often requiring inotropic support and fluid resuscitation. End organ damage typically seen includes elevated troponins, abnormal renal and liver function, and elevated lactic acid. Other inflammatory markers including ESR, CRP, and ferritin will also be elevated along with persistent fever exhibited by patients [2, 5]. The CDC has released a report of a similar hyperinflammatory syndrome observed in adults referred to as multisystem inflammatory syndrome in adults (MIS-A). This syndrome can masquerade as other types of shock but is treated entirely different emphasizing the clinical importance of prompt and correct identification of this disease process. We report two cases that fulfill the CDC criteria for MIS-A including significant cardiac dysfunction and prior history of COVID-19 pulmonary infection. At the time these cases were hospitalized (August 2020) the predominate variant in Wisconsin was beta; with the three most prevalent variants identified as: B.1.1.164, B.1.139, and B.1.2. SARS-CoV-2 vaccinations were not available at that time.

Treatment of MIS-C has been similar to treatment of Kawasaki’s disease [3] and a majority of children with MIS-C have received immunoglobulin (IVIG) (80.5%), steroids (62.8%), and a smaller percentage receiving immune modulators such as tocilizumab or anakinra (22.6%) [2]. In the most recent report from the CDC on 16 patients with MIS-A, seven (43.8%) received IVIG, 10 (62.5%) received steroids and two (12.5%) received tocilizumab [5]. Coronary artery aneurysms (CAA) are one of the hallmark signs of Kawasaki disease and have been identified in MIS-C. Neither of our patients had obvious evidence of CAA on TTE imaging or CT but given the suspicion for the syndrome and possibility aspirin was given to both patients. Patient 1 had mild thrombocytopenia that resolved and patient 2 developed reactive thrombocytosis to a platelet count of 538 × 10^9^/L. Neither had documented evidence of thrombotic disease. The American College of Rheumatology have antiplatelets guidelines which recommend low dose aspirin (3–5 mg/kg/day) should be used in patients until normalization of platelet count and confirmation of normal CAA > 4 weeks out from diagnosis [3].

There is emerging data on the cardiovascular manifestations of SARS-CoV-2, not only in the acute phase but also during the convalescent and chronic phase [6, 7]. A recent report by Puntmann et al [8], showed that even in patients who recently recovered from mild COVID-19 illness, up to 78% had abnormal cardiac findings indicative of ongoing myocardial inflammation independent of preexisting conditions, severity of initial COVID-19 symptoms, course of acute illness, and time from original diagnosis. The cause of SARS-CoV-2 cardiac involvement remains unclear and might be related to the presence of multiple co-morbidities in older patients with severe COVID-19 pulmonary disease and ARDS, direct viral-mediated toxicity, similar to what was observed in SARS [9], endothelial damage and thrombosis, cytokine-induced myocardial dysfunction [10] or dysregulation of the renin–angiotensin–aldosterone system [10, 11]. Gheblawi, noting the strong affinity of SARS-CoV-2 for the ACE2 receptors, suggests virus binding to ACE2 may lead to downstream loss of function of ACE2 with resultant myocardial dysfunction [11]. The late cardiac manifestations such as those seen in children [2, 4] and adults [5] seem most closely related to a post-infectious hyperinflammatory state and treatment with anti-inflammatories usually results in prompt and complete resolution of the cardiac findings [3].

Our case report contributes to the small number of reported cases on MIS-A and demonstrates the benefit of prompt anti-inflammatory treatments of IVIG, corticosteroids, and tocilizumab, and the generally positive outcome for complete recovery of cardiac function.

## Data Availability

Data sharing is not applicable to this article as no datasets were generated or analyzed during the current study.

## Abbreviations

MIS-A: Multi-inflammatory syndrome in adults
MIS-C: Multi-inflammatory syndrome in children
SARS-CoV-2: Severe acute respiratory syndrome coronavirus 2
ED: Emergency Department
NP: Nasopharyngeal
TTE: Transthoracic echocardiogram
LVEF: Left ventricular ejection fraction
IVIG: Intravenous immunoglobulins
CT: Cycle-threshold
CAA: Coronary artery aneurysms
